# Formulary for the Preparation and Employment of Several New Medicines; Such as Morphia, Codeine, &c.

**Published:** 1835-07-01

**Authors:** 


*Formulaire pour la Préparation et l'Emploi de plusieurs Nouveaux Médicaments; tels que la Morphine, la Codéine, l'Acide Prussique, la Strychnine, la Vératrine, l'Ether Hydrocyanique, le Sulfate de Quinine, la Cinchonine, l'Emétine, la Salicine, le Brôme, l'Iode, l'Iodure de Mercure, le Cyanure de Potassium, l'Huile de Croton Tiglium, les Sels d'or, les Sels de Platine, le Chlore, les Chlorures de Chaux et de Soude, les Bi-carbonates Alcalins, la Grenadine, le Phosphore, l'Acide Lactique, l'Huile Volatile de Moutarde, &c. &c.* Par F. MAGENDIE. *Huitième édition, revue et augmentée.* A Paris, Janvier, 1835.

*Formulary for the Preparation and Employment of several new Medicines; such as Morphia, Codeine, &c.* By F. MAGENDIE. Paris, 1835. 12mo. pp. 438.

WE had originally intended, in reviewing the useful manual before us, to confine ourselves to the matter added in this edition; but we have been induced to depart from this our first design, partly by the fact of these additions being but few, and partly by the consideration that, as only a small proportion of our readers can be supposed to possess the seventh edition of the "Formulaire," it may be interesting to the majority, if we go farther back in our analysis. Omitting therefore, with one or two exceptions, everything contained in the first four editions, we have considered all the rest as fair booty, and accordingly proceed to present our readers with the *spolia opima* of our collation.

*Resin of the Nux Vomica.* A tincture is prepared of this substance, containing four grains in the ounce. It may not only be employed internally, but in frictions on atrophied or paralysed parts. This method is much used in Italy, says M. Magendie; and he has employed it a good deal himself of late, but thinks it useful to add ammonia, as in the following formula:

Take of Tincture of Nux Vomica, an ounce;
Concentrated Ammonia, two drachms.

It was found beneficial in the treatment of the cholera at Paris.

*Salts of Strychnine.* Strychnine, by being combined with acids, becomes more soluble, and more active. Thus, if the patient drinks lemonade, the strychnine which he takes will be more powerful. The subcarbonate alone is scarcely soluble. The author has found marked effects produced by doses of one twelfth of a grain of the sulphate in a case of paraplegia, and has lately obtained favourable results from the iodate, in several cases of paralysis supposed to be incurable.

*Morphia, and its Salts.* It appears from the researches of Séguin, Derosne, Sertuerner, Robiquet, Robinet, Pelletier, and

Couerbe, that opium is composed of, 1, a fixed oil; 2, Caoutchouc; 3, Gum; 4, Fecula; 5, Resin; 6, Lignin; 7, Morphia; 8, Narcotine; 9, Narceine; 10, Meconine; 11, Codeïne; 12, Meconic acid; 13, Another brown acid. But, according to Robiquet's late researches, there is neither codeic acid nor codeate of morphia in opium: what had been taken for them being either an acid salt, or hydrochlorate of morphia. Morphia may be obtained directly from the capsules of the indigenous poppy, and does not differ from that which is pro cured from opium, either in chemical or medical qualities.

M. Blondeau, in a communication to the Royal Academy of Medicine, affirms that, by fermenting an aqueous solution of opium by means of a little yeast, a viscous colouring matter is destroyed, which sets the morphia free, and furnishes it pure, with greater facility, and in greater quantity, than by any other known method.

M. Magendie prefers the sulphate to the acetate of morphia; partly because it is difficult to make a perfectly neuter acetate, and partly because the acetate, when placed in contact with water, divides into two parts, a soluble superacetate, and an insoluble subacetate. The sulphate of morphia may be obtained by dissolving morphia in diluted sulphuric acid, and allowing crystals to form by evaporation. The crystals resemble those of sulphate of quinine, but may easily be distinguished by their reddening, when treated with pure nitric acid.

M. Pelletier also prefers the sulphate to the acetate of morphia, because the former can always be obtained pure, while the latter is often mixed with narcotine; the reason being that narcotine is more soluble in alcohol than morphia. The acetate, too, is partly decomposed by the drying which it necessarily undergoes in order that it may be kept; but the sulphate being obtained by crystallization, no sulphate is ever formed.

The sulphate of morphia is soluble in twice its weight of distilled water.

*Pure Morphia* is less soluble than its salts, and less powerful in its effects on the animal economy. This is by no means a reason for neglecting it: on the contrary, it is sometimes very advantageous, precisely on account of its comparative weakness.

M. Magendie gives morphia most frequently in the form of a pill, and in the dose of a quarter or half a grain, with the view of procuring sleep, while the patient is suffering from chronic and painful diseases. He believes, (but states his belief with some hesitation,) that the sleep procured in this manner is more durable, and more complete, that when it is owing to the salts of morphia.

*The Salts of Morphia.* Magendie seems generally to begin with the syrup of the acetate of morphia, and, when the patients become accustomed to its action, he then uses the syrup of the sulphate. His solution of the acetate contains sixteen grains to an ounce of distilled water, with three or four drops of acetic acid, and a drachm of alcohol. The dose is from six to twenty-four drops.

Both the acetate and the sulphate of morphia may be given in quantities varying from a quarter of a grain to two or three grains within twenty-four hours; and the author has even administered them in the dose of four grains daily, without inconvenience. Yet it appears, from another passage, that M. Magendie believes a quarter of a grain of morphia to be equivalent to a grain of the extract of opium; for, in speaking of the extract of opium deprived of morphia, he says "On peut donner cet extrait par grains; il m'a paru que quatre grains n'équivalent point, pour l'activité, à un grain d'extrait aqueux ordinaire, et à $\frac{1}{4}$ de grain de morphine." (P. 66.) When the dose is pushed too far, the salts of morphia occasion nausea or even vomiting.

*Solution of the Citrate of Morphia.* The black drops, which have long been employed, consist of a vegetable acid (usually the citric or acetic,) combined with opium. The preparation called citrate of morphia, by Dr. Porter, of Bristol, contains, in addition, narcotine and all the other crystallizable components of opium. Magendie, therefore, employs a solution of sixteen grains of morphia and eight of citric acid in an ounce of distilled water, coloured with two drachms of tincture of cochineal. The citrate of morphia is incompatible with lime water, solution of ammonia, and other alkaline substances.

*Extract of Opium deprived of Morphia.* The process of obtaining morphia from opium never deprives it entirely of this alkali, and the remaining extract may therefore be used instead of common opium, but in larger doses, as above stated.

*New Principles discovered in Opium.* These are three in number, *narceine, meconine,* and *codeine:* they were all discovered in 1832.

Narceine and meconine do not appear to have any effect upon the animal economy, and at present, therefore, can be considered only as chemical curiosities; though it must be confessed that the experiments cited by our author are neither numerous nor varied enough to settle the point.

Narceine is white and inodorous; its crystals are long and acicular; and it has a slightly bitter, and almost metallic, taste. It dissolves in 230 parts of boiling water, and 375 parts of cold water. It melts at 92° of the centigrade thermometer (197 $\frac{6}{10}$° of Fahr.) and is decomposed by a higher temperature The principal characters of narceine are those which are developed by acids. When concentrated, they decompose it entirely, especially when aided by heat. When diluted with half their weight of water, they produce remarkable changes of colour. Thus, at the instant the two substances come in contact, a beautiful blue is produced; and, if the water is absorbed by means of magnesia, or the chloride of lime, a rose colour is obtained. Hence M. Couerbe calls it the vegetable chameleon.

Codeine was discovered by M. Robiquet. He found that the muriate of morphia, prepared by Gregory's method, did not afford the quantity of morphia which it ought to have done. He suspected that it was impure, and found that its impurity was occasioned by a new substance, crystallizing in beautiful prisms, soluble in water, ether, and alcohol, and possessing very active alkaline properties. This was codeine.

This substance is insoluble in alkaline solutions; it combines with acids, and saturates them, forming salts which are precipitated by nutgalls. Moreover, nitric acid does not redden it, and the chloride of iron does not take any particular colour from this substance.

A grain of codeine, injected into the jugular vein of a middle-sized dog, caused profound sleep; but the animal recovered. A grain of the hydrochlorate of codeine, used in the same way, caused death.

M. Magendie has given codeine to a considerable number of patients at the Hôtel-Dieu; and he finds that a grain, administered at once or at twice, is sufficient in certain cases to produce sleep, which in general is calm and undisturbed, and is not followed the next day by drowsiness or a sense of weight in the head; effects frequently produced by morphia. A grain of codeine seems to be equivalent to half a grain of pure morphia. Two grains of codeine have more than once excited nausea, and even vomiting. The hydrochlorate is much stronger. Two grains generally cause not only sleep, but vertigo, nausea, and even vomiting. But, on the other hand, when this dose was given, M. Magendie has seen facial and sciatic neuralgiæ, which had resisted every other remedy, disappear as if by enchantment.

Many patients too, who had exhausted the narcotic action of morphia and its salts, have experienced the most satisfactory effects from codeine, taken alternately with the nitrate or the hydrochlorate of this vegetable base.

*The Muriate of Morphia and Codeine*, or Gregory's salt, partaking, as it does, of the virtues of morphia and codeine, may occasionally be substituted for either of them.

*Emetine.* Under this head our author mentions some experiments made by M. Chomel upon Violine, which is nothing more than emetine procured from the root of the violet. He gave from six to twelve grains in three doses, to nine patients. In six of them vomiting was produced; two only experienced a slight purging. One of these persons, who was suffering from diarrhœa, was cured by the third dose; and thus, in these two cases, violine produced neither vomiting nor purging. It was administered pure to two other patients, in the dose of three grains and a half, taken at three times. In one case it caused no vomiting, but only two liquid stools. The second patient had one fit of vomiting; and a third dose of two grains produced neither vomiting nor purging.

*Veratrine.* Four substances have been discovered in the seed of the Veratrum Sabadilla; namely, veratrine, sabadilline, a gum resin, and veratrin. M. Magendie has several times treated tic doloureux of the face with success, by sprinkling small blisters applied over the course of the affected nerves with one or two grains of veratrine, and repeating this dose every four or five days. He employs the same remedy, and with the same advantage in cases of paralysis of the face. The "pommade de veratrine" which he gives contains only four grains of this potent alkaloid in the ounce, and not twenty or forty, as was recommended by a certain author, who shall be nameless. It may be used externally in cases of chronic rheumatism, anasarca, and gout. When it is desirable to administer veratrine internally, the following formulæ will be useful:

Take of Veratrine, four grains;
Alcohol, an ounce.

The dose is from ten to twenty-five drops in any suitable vehicle.

Take of Sulphate of Veratrine, one grain;
Distilled water, two ounces.

The dose is a teaspoonful in an ounce or two of sugared water.

Veratrine uncombined is insoluble in water.

Veratrine (the discovery of which is due to M. Couerbe,) has not yet been used. M. Magendie has tried some experiments with sabadilline upon animals and men, but sees no reason as yet to prefer it to veratrine, under any circumstances.

*Prussic Acid.* M. Magendie narrates a lamentable occurrence which took place some years since in a Parisian Hospital. Our author's hydrocyanic syrup is the one commonly used; but, in the hospital, the hydrocyanic syrup of the codex is employed, which is very much stronger. Seven epileptic patients took, at the same time, about two drachms and six grains of the hydrocyanic syrup of the codex, and in three quarters of an hour they were no more. The moral which our author draws from this frightful occurrence is that everybody ought to use his syrup; whereas we would rather suggest, that every French doctor ought to be intimately acquainted with the Codex. The physician who has but a slight knowledge of the pharmacopœia of the country in which he lives, will often find that his prescriptions are ridiculously feeble, and sometimes that they are destructively active; and will soon confess that the unpraised but necessary accomplishment which he wants, if not a branch of medical science in name, is one in reality.

*Cyanuret of Potassium.* This remedy has been sometimes substituted for prussic acid, on account of the facility with which the latter is decomposed. The cyanuret is dissolved in eight times its weight of distilled water, being thus transformed into hydrocyanate of potash. When dissolved in this proportion it may be called medicinal hydrocyanate of potash. The following formulæ contain these remedies.

*Pectoral Mixture.*

Take of Medicinal Hydrocyanate of Potash, a drachm;
Distilled water, a pound;
Lump sugar, an ounce and a half.

The dose is a tablespoonful morning and evening.

*Pectoral Potion.*

Infusion of Ground Ivy, two ounces;
Medicinal Hydrocyanate of Potash, fifteen drops;
Syrup of Mallow, an ounce.

The dose is a teaspoonful every three hours.

*Potion containing the Cyanuret of Potassium.*

Lettuce water, two ounces;
Cyanuret of Potassium, from half a grain to two grains;
Syrup of Marsh Mallow, an ounce;

The dose is a tablespoonful every two hours.

*Syrup of Hydrocyanate of Potash.*

Simple syrup, a pound;
Medicinal Hydrocyanate of Potash, a drachm.

This is employed, instead of the ordinary syrups, as an addition to pectoral potions.

*Hydrocyanic Ether.* A patient, suffering from convulsive cough, took six drops of this remedy daily in a gum mixture. The effects were satisfactory, and no complaint was made of the penetrating and disagreeable smell of the ether. But, although several other patients who took this medicine at the Hôtel-Dieu were benefited by its use, M. Magendie was obliged to discontinue its employment, on account of the insurmountable disgust caused by the smell of the mixture.

*Iodine and Hydriodate of Potass.* M. Magendie has not yet seen any instances of the bad effects produced by over doses of iodine, but cites many witnessed by other physicians. The majority are certainly in the right in this instance, and we believe that our author stands alone in believing in the "innocuité de cette nouvelle substance." Iodine is a valuable, a most valuable remedy, but its effects must be watched with scrupulous caution. Its power in curing syphilitic eruptions is singularly great: for this purpose we give half a grain or a grain a day, dissolved in water, with a few grains of the hydriodate of potash. Our author prefers the hydriodate of potash alone dissolved in distilled water, in the proportion of thirty-six grains to an ounce; and he has given one, two, and even three, ounces of this solution in a day, without any unpleasant consequences.

Scorbutic swellings of the gums have been cured in a few days by half a drachm of this solution, taken daily. In these cases it probably acted, as it does in goitre, by contracting the ultimate ramifications of the vascular system. It is with the same view that our author employs it in hypertrophy of the ventricles of the heart. This method, however, was not successful at the Salpétrière, where the patients are old; but has been more effective in young persons at the Hôtel-Dieu, and in private practice. When the hypertrophy is accompanied, as it often is, by acceleration of the movements of the heart, M. Magendie adds digitalis, in the following manner:

*Atrophic Solution.*

Distilled lettuce water, eight ounces;
——— orange flower water, one drachm;
Ioduret of Potash, four drachms;
Tincture of Digitalis, one or two drachms;
Syrup of Mallow, an ounce and a half.

The dose is half an ounce, morning and evening, in a little water.

The following formulæ are extremely useful in chronic rheumatism and old syphilitic affections.

*Ioduretted Sarsaparilla.*

Decoction of Sarsaparilla, two pounds;
Ioduret of Potash, a drachm;
Syrup of Orange Peel, two ounces; to be taken daily, by a glassful at a time.

*Ioduretted Taraxacum.*

Decoction of Taraxacum, two pounds;
Ioduret of Potassium, half a drachm;
Syrup of Mint, two ounces.

To be taken daily, by a glassful at a time.

"I believe that I shall be useful to my brethren," says M. Magendie, "by pointing out to them a good way of curing scrofulous ophthalmia in a short time; as this is a disease which the majority of therapeutic agents fail in combating, even the most active ones, such as blisters, and a seton in the back of the neck." (P. 241.)

*Ioduretted Collyrium.*

Rose water, six ounces;
Ioduret of potassium, twenty-four grains;
Iodine, one or two grains.

To be used four times a day.

He has rarely seen scrofulous ophthalmia, even when complicated with ulceration of the conjunction and the cornea, resist this remedy for more than a month; of course, it is to be combined with appropriate internal remedies and regimen.

He sometimes adds morphia to the solution.

*The Iodate of Strychnine* is one of the most active salts known; a grain is sufficient to kill a large dog, with symptoms of tetanus.

M. Magendie has given it to several patients with a success which far surpassed his hopes. They were suffering from paraplegia, of long standing, and, having exhausted all known remedies, were reputed incurable. He has always administered it in pills, each containing an eighth of a grain; beginning with one every morning and evening, and increasing the dose until eight were taken in twenty-four hours. But in this, as in all the preparations of strychnine, the greatest circumspection is necessary.

*Ioduret of Sulphur.* An ointment, prepared with five parts of this substance to ninety-six parts of axunge, has been used for several years by M. Biett, in some cases of tuberculous affections of the skin.

*Bromine.* The effects of this remedy resemble those of iodine. M. Magendie gives the preparations of the former when iodine does not seem sufficiently effective, or when patients have become accustomed to its action. He uses it in scrofula, in amenorrhœa, and in hypertrophy of the ventricles. The following are his formulæ.

*Potion containing the Hydrobromate of Potash.*

Distilled Lettuce Water, three ounces;
Hydrobromate of Potash, twelve grains;
Syrup of Mallow, an ounce.

To be taken in the course of twenty-four hours, by a tablespoonful at a time.

*Pills of Bromuret of Iron.*

Powdered Bromuret of Iron, twelve grains;
Conserve of Roses, eighteen grains;
Gum acacia, twelve grains;

To make twenty pills; two to be taken every morning and evening.

*Bromine Ointment.*

Axunge, an ounce;
Hydrobromate of Potash, or of Soda, thirty-four grains.

Half a drachm, or a drachm, is to be rubbed upon scrofulous swellings.

*Ointment of Bromuretted Hydrobromate of Potash.*

Pure axunge, an ounce:
Hydrobromate of potash, twenty-four grains;
Liquid bromine; from six to twelve grains.*

To be used in frictions.

*Mannite.* This is one of the substances first added in the present edition. It is obtained in the following manner: The manna of commerce, known by the name of manna in the tear, is treated with boiling alcohol; then filtered, and allowed to crystallize; the mannite is precipitated in small needles, beautifully white. Mannite has the purgative property of manna, without its nauseous smell. The dose for children is two drachms: half an ounce is too laxative.

*Solanine.* This alkali was discovered by M. Desfosses of Besançon in the Solanum nigrum and the Solanum Dulcamara; but other people, though they have repeated the experiments, have not found the alkali. Hence our author thinks it essential "que M. Desfosses voulût bien répéter ses expériences, afin de constater de nouveau le fait qu'il a avancé, ou bien d'indiquer à quelle circonstance il tient qu' à Paris on n'a pu obtenir de solanine." (P. 299.)

*Thridace.* This word is derived from θριδαξ, a lettuce, and is equivalent to Lactucarium, or Extractum Lactucæ. Under this head our author mentions that MM. Caventou and Boulay were not able to find any especial principle in the extract of lettuce that resembled morphia. (P. 344.) Dr. A. T. Thomson, on the other hand, tells us that a solution of lactucarium, when treated in the same manner as opium, discovers the presence of Morphia. (*Dispensatory*, 4th Edit., p. 382.)

*The Salts of Gold.* According to M. Chrestien (*Méthode*

* The original has *gros*, (p. 261.) but we have not followed it; as it is evidently a misprint for *grains*.

*iatraleptique,* 2d Edit., p. 398 and 399.) "The muriate of gold is infinitely more active than corrosive sublimate, but is less irritating to the gums: when administered in the dose of the tenth of a grain daily, it caused considerable fever in one case." M. Chrestien remarks, that the agitation of the pulse, that is to say, the frequency of the pulsations and the largeness of the beat (*développement de l'artère,*) always occurs when his method is employed, (i. e. when the salt of gold is rubbed in,) and particularly when the muriate is used.

These preparations are chiefly employed in syphilis: the dose of the muriate of gold and soda is from a fourteenth to an eighth of a grain daily; it is mixed with the powder of lycopodium, and rubbed upon the tongue and gums.

The following pills are used by M. Chrestien in scrofula, beginning with one, and increasing the dose to seven or eight a day.

*Pills of the Oxide of Gold.*

Take of Extract of the Bark of the Root of the Daphne Gnidium, two drachms;
Oxide of Gold, prepared with Potash, six grains.

To make sixty pills.

One grain of the muriate of gold and soda may be used instead of the six grains of oxide.

Dr. Niel, who has written on the employment of the preparations of gold, advises a particular method of using them, when the state of the tongue, or of the inside of the mouth, does not allow frictions to be applied to these parts. His method is as follows:

The cutis is to be laid bare on one side of the neck by a small blister, and the part is to be dressed morning and evening with a mixture composed of a grain of axunge and a grain of gold, divided by mercury; at the same time, the oxide of gold is to be given in the form of a pill, and in the dose of a grain daily. At the end of a week, the doses of divided gold and of the oxide are each augmented by half a grain. At the end of a fortnight, as the sore begins to heal, it is stimulated with blister-ointment, and instead of the divided gold, the tenth part of a grain of the muriate of gold and soda is substituted, with a little lard. The application of this ointment causes itching, but little irritation, and the quantity of muriate used in dressing may be successively carried to an eighth and even a sixth of a grain. During the whole period of the external treatment, the internal use of the oxide of gold is continued; and this treatment is to be continued for some time after the disappearance of all the syphilitic symptoms.

The second case related by M. Niel relates to a person in whom the use of the muriate of gold and soda, in the dose of the tenth of a grain rubbed upon the tongue, caused such violent irritation, that it was necessary to substitute some other manner of employing the preparations of gold. Therefore, after having applied a slip of blistering plaster upon each side of the neck, the sores were dressed, first of all with a slight layer of the following mixture:

Take of Gold divided by means of mercury, a drachm;
Axunge, an ounce.

When the sores were beginning to dry up, this ointment, with the oxide, gave way to one made with the muriate, in the following proportions:

Take of Muriate of Gold and Soda, ten grains;
Axunge, half an ounce.

The employment of these remedies was continued for four months, and the cure was perfect.

Dr. Simoneau, a physician of Florensac, having applied a seton to the back of the neck of a patient, who had serious ulcerations in his mouth, hit upon the happy idea of dressing the sore morning and evening with the muriate of gold, mingled with a little fat. This mode of treatment, which is analogous to the one employed by M. Niel, was perfectly successful.

*The salts of Platinum* are prepared in the same way as those of gold. M. Cullerier has tried the hydrochlorate of platinum and soda, and its effects do not differ from those of the hydrochlorate of gold and soda.

*Lactic acid.* This is one of the substances added in the present edition. The existence of lactic acid was long doubtful: though acknowledged by several distinguished chemists it was denied by others not less celebrated, who believed the lactic and acetic acids to be identical. This question is now decided in the affirmative, to the great advantage of therapeutics; as it appears very probable to our author that this acid will become a useful medicine.

*Method of obtaining Lactic acid.* It is extracted either from milk, or from the juice of beet-root. If the latter is to be used it is left to itself in a vessel of which the temperature must always be kept between 77° and 85° of Fahrenheit. In a few days, a tumultuous movement, called the viscous fermentation, takes place in the whole mass, and hydrogen gas, mingled with carburetted hydrogen gas, is disengaged in great abundance. When the liquid has regained its former fluidity, and the fermentation is over, which is generally the case in about two months, it is to be evaporated to the consistence of a syrup; the whole mass is then traversed by a number of crystals of mannite, which, after being washed with a small quantity of cold water, and then pressed, are perfectly pure; the mass contains, moreover, a sugar, which presents all the properties of grape sugar. The product of evaporation is treated with alcohol, which dissolves the lactic acid, and precipitates a number of substances, which have not yet been examined; the alcohol extract is mixed with water, which leaves a fresh deposit; and the liquor is then saturated with carbonate of zinc, by which a more abundant precipitate is caused than either of the others. After concentration, the lactate of zinc crystallizes; it is collected, and heated with water, to which is added animal charcoal, previously washed with hydrochloric acid; it is filtered while boiling, and the lactate of zinc separates in perfectly white crystals; these are again washed with boiling alcohol, in which they are insoluble. They are then successively treated with barytes and sulphuric acid, and the lactic acid thus obtained is concentrated in vacuo. Lastly, by shaking it with sulphuric æther, which dissolves it, some traces of flaky matter are separated. (*Annales de Chimie et de Physique*, Avril, 1833.)

A large quantity of milk, suffered to ferment, and treated in the same manner, also furnishes lactic acid. M. Corriol has recognised its presence in the aqueous infusion of nux vomica.

*Physical and Chemical Properties of Lactic Acid.* When concentrated in vacuo till it ceases to lose water, lactic acid is a colourless liquid of the consistence of syrup, and whose density, at the temperature of 68½° of Fahrenheit, is 1,215. It is inodorous; its taste is exceedingly acid, and comparable to that of the most powerful vegetable acids; when exposed to the atmosphere, it attracts moisture from it: water and alcohol dissolve it in any proportion.

One of its most remarkable properties, and one which the physician is especially concerned in knowing, is the facility with which it dissolves phosphate of lime, particularly that of the bones.

*Method of using Lactic Acid.* As lactic acid is an agent in the solution of food in the stomach, M. Magendie thought that it might be advantageously employed in cases of dyspepsia, or of simple debility of the digestive organs, and has found its effects very satisfactory. He gives it in the form of lemonade or lozenges, as in the following formulæ.

*Lactic Lemonade.*

Take of Liquid Lactic Acid, from one to four drachms;
Common Water, a pint;
Simple Syrup, two ounces.

*Lactic acid lozenges.*

Take of Pure Lactic Acid, two drachms;
Powdered sugar, an ounce;
Gum Tragacanth, q. s.
Volatile Oil of Vanilla, four drops.

To be made into lozenges of half a drachm each, which are to be kept in a well-stopped vessel. Six of these lozenges may be taken within twenty-four hours, without inconvenience.

The facility with which lactic acid dissolves the phosphate of lime makes it reasonable to try this acid in cases of white gravel, or phosphate of lime. M. Magendie has not yet had an opportunity of doing this, but intends it. He has also begun a series of clinical experiments with the lactates of soda, of potash, &c., but has not yet lighted upon anything worth publishing. He recommends these salts, however, to the attention of physicians.

*Volatile Oil of Black Mustard Seeds.* This oil, when diluted with an equal weight of alcohol, of the specific gravity of 0,828, is an excellent rubefacient, and its action is almost instantaneous. If a part is rubbed with it for a few minutes, *phlyctenæ* are soon formed, resembling those created by blisters.

It is needless to give our opinion of the work before us; its reputation is European, and the physician who has succeeded in diffusing a knowledge of the new remedies over the civilized world, does not require our feeble praise. It would be easy, of course, to remark that some of the medicines are of doubtful merit, and that the hydrocyanic ether, which only one patient could take, and the solanine, which only one chemist could make, might, with a few others, be omitted. But, in the matter of remedies, the old rule holds good, *præstat copiâ quam penuriâ premi;* and we would admit on slighter grounds than we would expel. The only important omission that we have observed, and it is an unaccountable one, is of creosote.

Without going quite so far as our author, who asks what practitioner would now give cinchona in powder or extract, in preference to the sulphate of quinine, or salicine, we would affirm that an accurate acquaintance with the new remedies becomes every day more necessary; and we would observe, in addition, that a physician must be far indeed above or below instruction, if the study of this Formulary does not lend new vigour to the substance of his prescriptions, and new elegance to their form.

